# Iron supplementation to treat anaemia in adult critical care patients: a systematic review and meta-analysis

**DOI:** 10.1186/s13054-016-1486-z

**Published:** 2016-09-29

**Authors:** Akshay Shah, Noémi B. Roy, Stuart McKechnie, Carolyn Doree, Sheila A. Fisher, Simon J. Stanworth

**Affiliations:** 1 Nuffield Department of Anaesthetics, Level 2 John Radcliffe Hospital, Oxford University Hospitals NHS Foundation Trust, Oxford, UK; 2 Weatherall Institute of Molecular Medicine, University of Oxford, Oxford, UK; 3 Systematic Review Initiative, NHS Blood & Transplant, Oxford, UK; 4 NHS Blood and Transplant, Oxford University Hospitals NHS Foundation Trust, John Radcliffe Hospital, Oxford, UK; 5 Radcliffe Department of Medicine, University of Oxford, Oxford, UK

**Keywords:** Anaemia, Transfusion, Red blood cell, Iron, Haemoglobin, Meta-analysis

## Abstract

**Background:**

Anaemia affects 60–80 % of patients admitted to intensive care units (ICUs). Allogeneic red blood cell (RBC) transfusions remain the mainstay of treatment for anaemia but are associated with risks and are costly. Our objective was to assess the efficacy and safety of iron supplementation by any route, in anaemic patients in adult ICUs.

**Methods:**

Electronic databases (CENTRAL, MEDLINE, EMBASE) were searched through March 2016 for randomized controlled trials (RCT)s comparing iron by any route with placebo/no iron. Primary outcomes were red blood cell transfusions and mean haemoglobin concentration. Secondary outcomes included mortality, infection, ICU and hospital length of stay, mean difference (MD) in iron biomarkers, health-related quality of life and adverse events.

**Results:**

Five RCTs recruiting 665 patients met the inclusion criteria; intravenous iron was tested in four of the RCTs. There was no difference in allogeneic RBC transfusion requirements (relative risk 0.87, 95 % confidence interval (CI) 0.70 to 1.07, *p* = 0.18, five trials) or mean number of RBC units transfused (MD -0.45, 95 % CI -1.34 to 0.43, *p* = 0.32, two trials) in patients receiving or not receiving iron. Similarly, there was no difference between groups in haemoglobin at short-term (up to 10 days) (MD -0.25, 95 % CI -0.79 to 0.28, *p* = 0.35, three trials) or mid-term follow up (last measured time point in hospital or end of trial) (MD 0.21, 95 % CI -0.13 to 0.55, *p* = 0.23, three trials). There was no difference in secondary outcomes of mortality, in-hospital infection, or length of stay. Risk of bias was generally low although three trials had high risk of attrition bias; only one trial had low risk of bias across all domains.

**Conclusion:**

Iron supplementation does not reduce RBC transfusion requirements in critically ill adults, but there is considerable heterogeneity between trials in study design, nature of interventions, and outcomes. Well-designed trials are needed to investigate the optimal iron dosing regimens and strategies to identify which patients are most likely to benefit from iron, together with patient-focused outcomes.

**Trial registration:**

PROSPERO International prospective register of systematic reviews CRD42015016627. Registered 2 March 2015.

**Electronic supplementary material:**

The online version of this article (doi:10.1186/s13054-016-1486-z) contains supplementary material, which is available to authorized users.

## Background

Anaemia is common in critically ill patients and is associated with adverse outcomes [1, 2]. Observational studies have shown that anaemia affects 60–80 % of patients admitted to critical care and is present at critical care discharge in 75 % of patients [3, 4]. The implementation of restrictive red cell transfusion policies is likely to compound the observed high prevalence of anaemia [5]. Allogeneic red blood cell (RBC) transfusion has been the mainstay of treatment for critical illness anaemia, although studies suggest that patients who receive allogeneic RBC transfusion are at increased risk of mortality, ischaemic complications, delayed wound healing, multi-organ dysfunction and increased length of stay [6–9].

Interest has now focused on the identification of iron-deficiency as an aetiological factor contributing to the anaemia observed in critical illness. However, diagnosis of absolute iron-deficiency continues to be challenging in critical care. Critically ill anaemic patients typically display the hallmarks of anaemia of inflammation and iron-restricted erythropoiesis secondary to functional iron deficiency, absolute iron deficiency or iron sequestration [10]. As for other inflammatory conditions, hepcidin as a key regulator of iron homeostasis is considered to contribute to this functional iron deficiency in critically ill patients [10]. Iron supplementation, whether oral or intravenous (iv), is an effective treatment for patients with absolute iron deficiency; however, iron is essential for bacterial growth, and exogenous intravenous iron administration has been reported to be associated with increased risk of infection, raising significant safety concerns, particularly in this patient group [11].

To assess current knowledge of the role of iron supplementation in critical care, we performed a systematic review to evaluate the effect of iron supplementation on allogeneic RBC transfusion requirements and clinical outcomes (mortality, quality of life and infection) in this patient group. The safety of iron supplementation was also evaluated.

## Methods

Study methods were implemented according to a predefined protocol [12].

### Study identification

Trials were identified from database searches to 14 March 2016 of CENTRAL (The Cochrane Library Issue 2, 2016), MEDLINE (1946 onwards), Embase (1974 onwards), CINAHL (1937 onwards), PubMed (E-publications ahead of print only), Web of Science (1990 onwards) and the Transfusion Evidence Library (1950 onwards). We searched for ongoing trials using the trial registration websites World Health Organization International Clinical Trials Registry Platform and ClinicalTrials.gov. We did not restrict our search for trials by date, language, or publication status. The full search strategy is shown in Additional file 1.

### Eligibility criteria

To be included in this systematic review, the following criteria had to be met: (1) study design: randomized controlled trials; (2) patients: all patients admitted to any adult intensive care units (ICUs) or high-dependency units (HDUs); and (3) intervention: iron supplementation by any route (enteral, iv, intramuscular) versus placebo or no iron therapy; co-interventions were only included if they were present in both/all treatment arms. Trials including children, pregnant women and patients with chronic kidney disease were excluded [13, 14].

### Study selection and data extraction

Two reviewers (AS, NR) independently screened citations that met inclusion criteria and extracted data onto a pre-piloted data collection form. Disagreements were solved through discussion and if no resolution was found a third author (SS) adjudicated. Two reviewers (AS and SS) independently extracted study characteristics and outcomes, including study design, methodology, patient characteristics, iron supplementation strategies and outcome data. We sought unclear or missing data by contacting the authors of the individual trials and allowing them 28 days to respond.

### Study outcomes

The primary outcomes of this systematic review were: (1) requirement for allogeneic RBC transfusion; (2) mean number of RBCs transfused; and (3) mean haemoglobin concentration. Secondary outcomes were: (1) all-cause mortality; (2) in-hospital infection as defined in individual trials (e.g. confirmed/suspected diagnosis and antibiotic-free days); (3) mean ICU length of stay (LOS); (4) mean hospital LOS; (5) mean measurement of biomarkers of iron status, e.g. ferritin, erythrocyte zinc protoporphyrin (eZPP), transferrin saturation (Tsat) and serum iron; (6) morbidity scales (sequential organ failure assessment (SOFA) score and multiple organ dysfunction score (MODS)); and (7) health-related quality of life (HrQoL) (e.g. SF-36) and serious adverse events defined as “any untoward medical occurrence that at any dose results in death, is life-threatening, requires in-patient hospitalisation or prolongation of existing hospitalization, or results in persistent or significant disability/incapacity” [15]. We defined two time points: (1) short-term (up to 10 days) and (2) mid-term (the last measured time point in hospital or end of the trial).

### Risk of bias assessment

Study quality was judged using the Cochrane Collaboration tool for assessing risk of bias [16]. We assessed the following domains for each study: random sequence generation, allocation concealment, blinding of participants, personnel and outcome assessors, incomplete outcome data, selective reporting and other bias such as financial conflicts of interest. A three-point scale was used to determine low, high or unclear risk of bias.

### Data synthesis

Statistical analyses were performed using Review Manager [17]. Random effects models were used throughout due to the likely heterogeneity arising from different iron supplementation strategies used and different ICU settings. Where possible, continuous variables were reported as mean difference (MD) with 95 % confidence interval (CI) at follow-up time points as defined above. For outcomes which were deemed likely to be skewed in distribution, namely number of RBC transfusions and serum ferritin, reported mean and standard deviation were transformed to a natural logarithmic scale using the methods of Higgins et al. [18]. In one study that reported count data for the number of transfusions [19], raw data values were log transformed after adding a constant of 0.5 to each value due to the number of patients who received no transfusions. Dichotomous variables were reported as relative risk (RR) with corresponding 95 % CI. Heterogeneity was assessed using the *I*^2^ statistic [20]. In one trial with two intervention arms, data were pooled across the two intervention arms and compared with the single control group for the main analysis [19].

### Subgroup and sensitivity analysis

Subgroup analyses were performed if there were at least two trials with comparable subgroups reporting the primary outcomes: (1) different iron preparations (enteral vs intravenous) and (2) use of co-interventions, e.g. erythropoietin. Sensitivity analyses for risk of bias were intended but were not carried out due to the limited number of included studies.

## Results

**Fig. 1:**
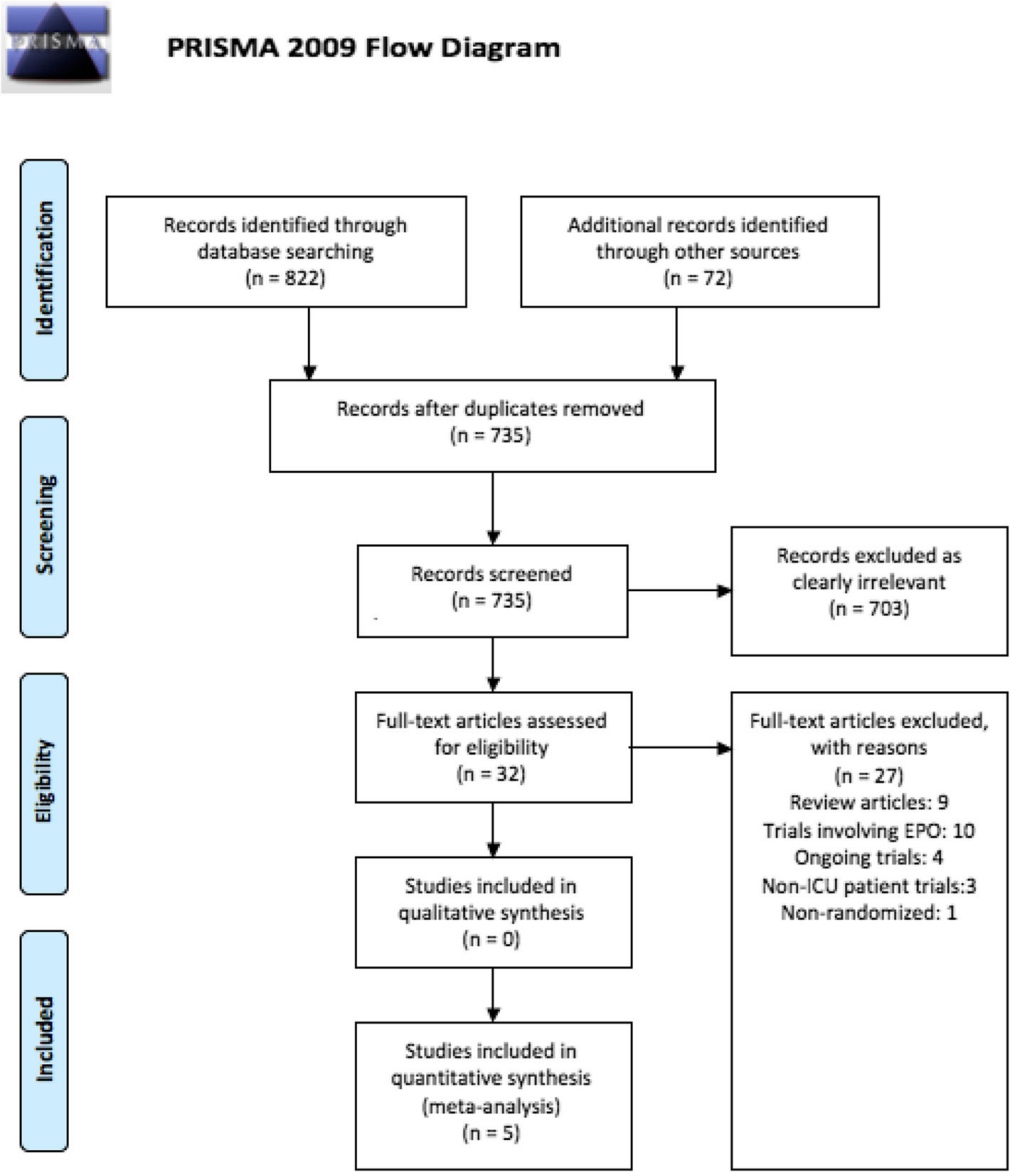
Preferred Reporting Items for Systematic Reviews and Meta-analyses (PRISMA) flow chart showing the selection of studies in this meta-analysis. *EPO* erythropoietin

Our search identified 894 references. Of these, 32 underwent full-text screening after exclusion by screening of titles, duplicates and abstracts (see Preferred Reporting Items for Systematic Reviews and Meta-Analyses (PRISMA) study flow diagram in Fig. 1). Five studies were included in the final analysis [19, 21–24]. One ongoing trial was identified [25].

### Description of included studies

Patient populations, trial interventions and outcomes are summarised in Table 1. The five studies included a total of 665 randomised participants of whom 368 received iron and 297 received no iron/placebo. Four trials were carried out in surgical ICUs (cardiothoracic, trauma, general surgical, burns and neurosurgical) and one trial was carried out in a mixed ICU that included medical, surgical and trauma patients. Two trials compared intravenous iron to intravenous placebo, one trial compared intravenous iron to no iron (control) and one trial compared oral iron to oral placebo. A fifth trial included three arms: (1) intravenous iron and oral placebo, (2) oral iron and intravenous placebo, and (3) oral and intravenous placebo. Co-interventions were given in two trials: ascorbic acid, cyanocobalamin and folic acid [22] or folic acid only [24]. All trials reported different dosing regimens as described in Table 1.

**Table 1:** Characteristics of included studies (*n* = 5)

	Study									
	Garrido-Martin et al. (2012) [19]		Madi-Jebara et al. (2004) [21]		Pieracci et al. (2009) [22]		Pieracci et al. (2014) [23]		van Iperen et al. (2000) [24]	
	Iron	No iron	Iron	No iron	Iron	No iron	Iron	No iron	Iron	No iron
Randomised, *n*	iv 71, oral 73	66	40	40	97	103	75	76	12	12
Analysed *n*	iv 54, oral 53	52	40	40	97	103	75	75	12	12
Age, mean (SD/range)	iv 65 (11) oral 65 (10)	65 (12)	59.1 (9.1)	55.3 (9.5)	55.7 (1.9)	58.2 (1.7)	41.6 (18–83)	40.4 (18–87)	67 (49–89)	69 (45–80)
Male, %:	iv 70.3 % oral 71.7 %	76.9 %	90 %	90 %	50.5 %	46.6 %	77.3 %	60.5 %	66.6 %	66.6 %
ICU setting	Cardiothoracic		Cardiothoracic		General surgical, burns, neurosurgical		Trauma		Mixed (surgical, medical, neurological, trauma)	
Inclusion criteria	Age >18 years old, elective cardiac surgery under extracorporeal circulation, no previous anaemia, susceptible to treatment, no preoperative blood transfusion, able to complete all study visits as per protocol, able to provide written consent		Elective cardiac surgery with CPB, post-pump Hb 7 –10 g.dL^-1^		General surgical, burn, neurosurgical ICUs, age >18 years, Hb <13 g.dL^-1^ prior to enrollment, <72 hours from hospital admission, current tolerance of enteral medication, expected ICU LOS >5 days		Admitted to ICU with trauma, Hb <12 g.dL^-1^, Age >18 years, <72 hours from ICU admission, expected ICU LOS >5 days		Hb <11.2 g.dL^-1^, <12.1 g.dL^-1^ if cardiac disease, age >18 years, expected ICU LOS >7 days, informed consent from patient or relative	
Exclusion criteria	Elective cardiac surgery without exclusion criteria, fibrinolytic therapy 48 hours prior to CPB, impaired renal function (CrCl <50 mls.min^-1^), previous surgery for IE, repeat surgery, pregnant or lactating, active gastrointestinal bleeding, B12 deficit, ferropenic anaemia, asthma or allergy, active infection, included in another study, hepatic disease, history of allergy to iron, unlikely to adhere to protocol follow up		Intra-operative blood transfusion, post-operative haemodynamic instability, ejection fraction <40 %, chronic kidney disease, hypothermic bypass, hypersensitivity to iron		Active bleeding, chronic inflammatory conditions, end-stage renal disease, haematologic disorders, macrocytic anaemia, current use of EPO, pregnancy, prohibition of RBC transfusion, imminent death, co-enrollment in another trial		Active haemorrhage, iron overload (serum ferritin >1000 ng.mL^-1^), conditions associated with iron overload e.g. haemachromatosis, active infection, chronic inflammatory conditions, pre-existing haematological disorders, macrocytic anaemia, current/recent (within 30 days) use of immunosuppression, use of EPO within 30 days, pregnancy or lactation, prohibition of RBCs, imminent death, history of allergy to iron		Pregnancy, iron deficiency anaemia (ferritin <50 ug.L^-1^), vitamin B12 deficiency (<160 pmol.L^-1^), recent use of cytostatics or recent radiotherapy, life expectancy <7 days, chronic renal failure, prior use of EPO	
Intervention(s)	(1) Iron-hydroxide sucrose complex, iv (Venofer; Uriach Laboratory) 3 doses of 100 mg/24 hours during pre- and post-hospitalisation, and 1 pill/24 hours of oral placebo during the same period and during 1 month after discharge (2) Ferrous fumarate (105 mg of iron) 1 pill/24 hours orally pre- and postoperatively and during 1 month after discharge, and intravenous placebo while hospitalised		i) Iron, iv (Venofer; Luitpold Pharmaceuticals) 200 mg/day to reach total iron deficit + s/c placebo ^(a)^		Enteral ferrous sulphate 325 mg (oral solution or capsule) (Rockwell Compounding Inc.) thrice daily until hospital discharge. Co-intervention: ascorbic acid 500 mg thrice daily, cyanocobalamin 1 mg, folic acid 1 mg		Iron sucrose, iv (Venofer; Luitpold Pharmaceuticals) 100 mg thrice weekly for up to 6 doses or until ICU discharge		Iron saccharate, iv (Venofer; Vifor) 20 mg and iv folic acid 1 mg daily from day 1–14 ^(b)^. Co-intervention: folic acid 1 mg daily	
Comparator	Oral and iv placebo pre-operatively and postoperatively following same protocol.		Placebo - s/c and iv (0.9 % saline).		Oral placebo, same schedule as intervention protocol. Co-intervention: ascorbic acid 500 mg thrice daily, cyanocobalamin 1 mg, folic acid 1 mg		Placebo, iv (100 mL of 0.9 % saline) similar dosing schedule to intervention		No iron. Co-intervention: folic acid 1 mg daily	
Reported outcomes (follow-up time points, days)	• Hb concentration (baseline, operating room entry (day 7), exit operating room, ICU admission, ICU discharge, postoperative day10 and day 30 post-hospital hospital discharge) • Immature reticulocyte fraction, reticulocyte count, serum ferritin (day 1, postoperative day 10 and day 30 post-hospital discharge) • RBC transfusion (number of patients transfused, location of transfusion, mean number of units)		• Hb concentration (day 0, day 1–5, day 15, day 30) • Reticulocyte counts (day 1, day 5, day 15, day 30) • Serum ferritin (day 0, ay 5, day 15) • RBC transfusion • Mortality		• Difference in Hct (baseline, days 7, 14, 21 and 28) (primary outcome) • Serum iron, serum ferritin, eZPP (baseline, day 7, day 14, day 21, day 28) • RBC transfusion • Estimated blood loss per study day • Nosocomial infection • Antibiotic days • Adverse outcomes - gastrointestinal upset • ICU and hospital LOS • Mortality		• Number of total doses of study drug received • Hb concentration (daily) • Serum iron, serum ferritin, serum Tsat, eZPP (baseline, day 7, day 14) • RBC transfusions • Transfusion-free days • Nosocomial infection and type • Antibiotic exposure • ICU and hospital LOS • Mortality		• Hb concentration (days 0, 7, 14, 21) • Reticulocyte count, sTfR (days 0, 7, 14, 21) • Serum EPO (days 0, 2, 6, 10, 21) • Serum iron, transferrin, Tsat, ferritin, eZPP, CRP (days 0, 10, 21) • Mean blood loss (day 0–21) • RBC transfusion (mean number of units) (day 0–21) • ICU LOS • Mortality	

^a^In a second intervention arm, patients received intravenous iron and recombinant-human erythropoietin (EPO) (300 IU/kg) subcutaneously (s/c) on day 1; this treatment arm was not included in this review because the co-intervention was not matched in the control group. ^b^In a second intervention arm, patients received intravenous iron and EPO alfa (300 IU/kg) s/c on days 1, 3, 5, 7, 9; this treatment arm was not included in this review because the co-intervention was not matched in the control group. *ICU* intensive care unit, *Hb* haemoglobin, *Hct* haematocrit, *Tsat* transferrin saturation, *sTfR* soluble transferrin receptor, *eZPP* erythrocyte zinc protoporphyrin, *LOS* length of stay, *CBP* cardiopulmonary bypass, *RBC* red blood cell, *CrCl* creatinine clearance, *IE* infective endocarditis

### Quality assessment and Risk of Bias

**Fig. 2:**
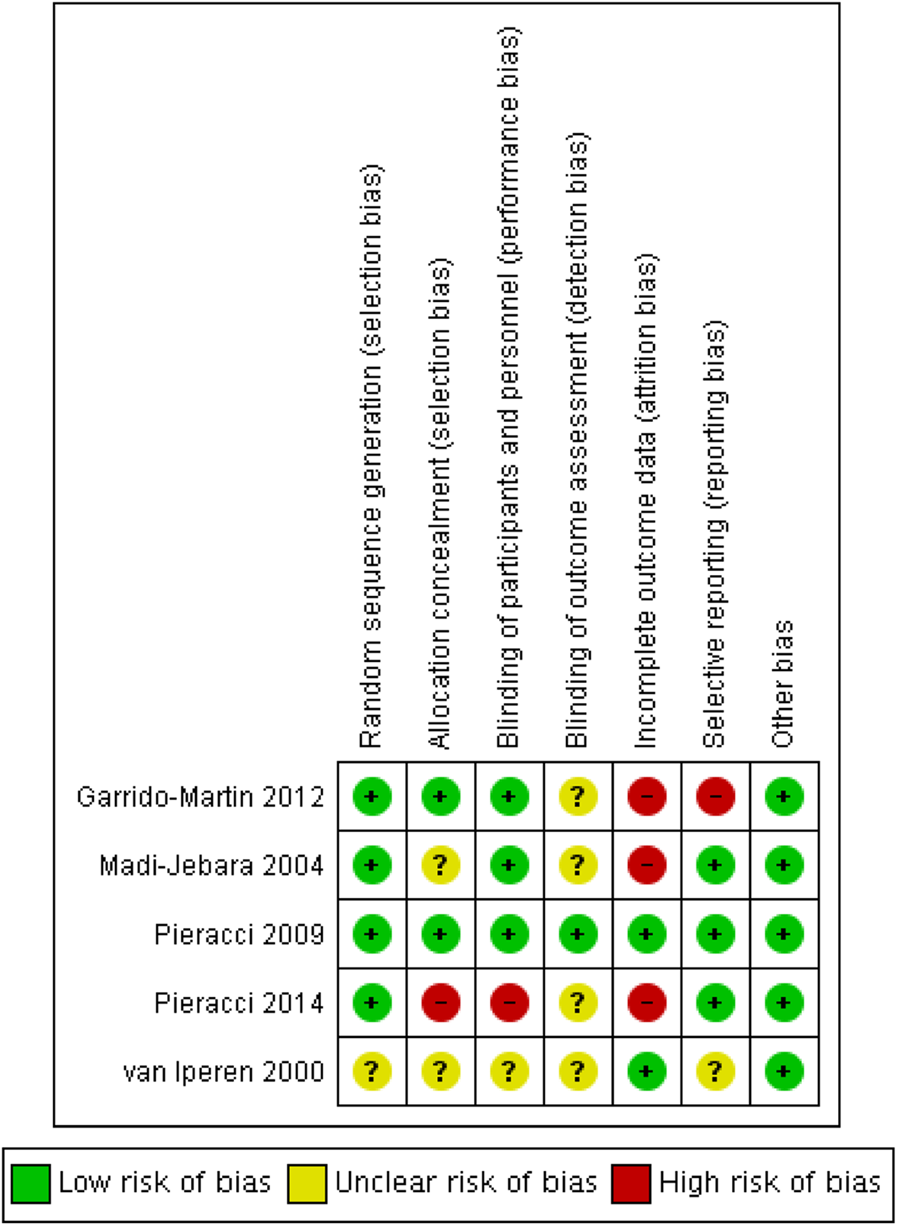
Risk of bias summary showing review authors’ judgements about each risk of bias item for each included study

The risk of bias in individual trials is shown in Fig. 2. Only one trial had low risk of bias in every domain [19]. Three trials reported high levels of attrition of randomized participants. In one trial, only participants who received all six possible doses of the study drug were analysed for haemoglobin and other markers of iron status (46/75 participants in the intervention groups and 48/75 participants in the control group) [23], in the second trial, 17 participants in the intervention group and 7 participants in the control group were excluded from the final analysis [19] and in the third trial only patients who had not received a blood transfusion were analysed [21].

## Effect of interventions

### Allogeneic red blood cell transfusion

**Fig. 3:**
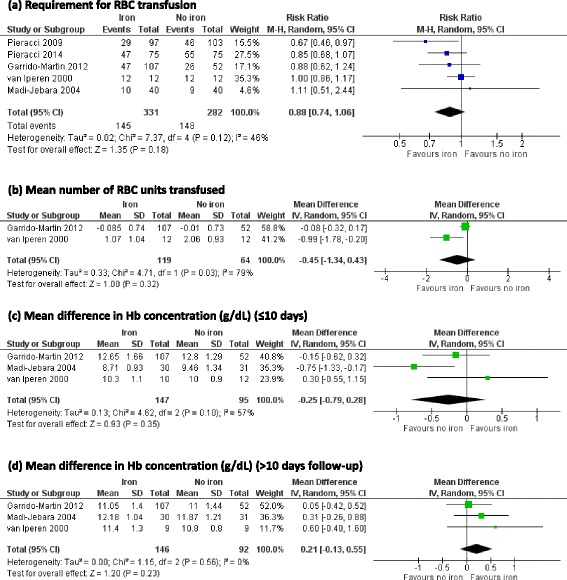
Effect of iron supplementation, by any route, on primary outcomes **a** requirement for RBC transfusion, **b** mean number of RBC units transfused and mean difference in Hb concentration at **c** short-term follow-up (upto 10 days) and **d** medium-term follow-up (last measured time point in hospital or end of the trial).* RBC* red blood cell, *Hb* haemoglobin, *CI* confidence interval, *M-H* Mantel-Haenszel test, *IV* inverse variance

**Fig. 4:**
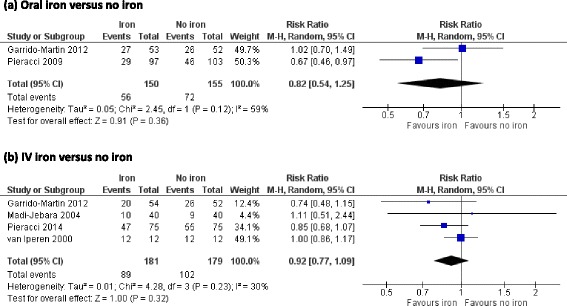
Subgroup analysis: effect of iron supplementation by either intravenous or oral administration on allogeneic red blood cell transfusion requirement **a** oral iron versus no iron, **b** intravenous iron versus no iron. *RBC* red blood cell, *CI* confidence interval, *M-H* Mantel-Haenszel test

All five trials reported on the number of patients who received an allogeneic RBC transfusion. In meta-analysis there was no evidence that iron supplementation to treat anaemia in critical care patients reduced allogeneic RBC transfusion requirements (RR 0.88, 95 % CI 0.74 to 1.06; *p* = 0.18, *I*^2^ = 46 %) (Fig. 3a). In subgroup analysis by route of administration there was no evidence of an effect of iron supplementation in patients who received oral iron or those who received intravenous iron (Fig. 4).

### Mean number of RBC units

Only two trials reported the number of RBC units received in both groups [19, 24]. After transforming to a log scale, meta-analysis of these two trials showed no evidence that iron supplementation reduced the mean number of allogeneic RBC units transfused (MD -0.45, 95 % CI -1.34 to 0.43, *p* = 0.32, *I*^2^ = 79 %) (Fig. 3b).

### Mean difference in haemoglobin concentration

Mean (and standard deviation (SD)) changes in haemoglobin concentration at short-term and mid-term follow-up time points was reported in three trials [19, 21, 24]. There was no significant difference in the mean haemoglobin concentration between patients who received iron and those who did not, either at short-term (MD -0.25, 95 % CI -0.79 to 0.28, *p* = 0.35, *I*^2^ = 57 %) (Fig. 3c) or mid-term follow-up (MD 0.21, 95 % CI -0.13 to 0.55, *p* = 0.23, *I*^2^ = 0 %) (Fig. 3d). In the two trials that did not report mean and SD, one trial reported no change in haematocrit concentration over a period of 28 days in both groups [22] and the other trial found no significant differences in haemoglobin concentration between groups at any follow-up time point over 14 days [23].

### In-hospital mortality

**Fig. 5:**
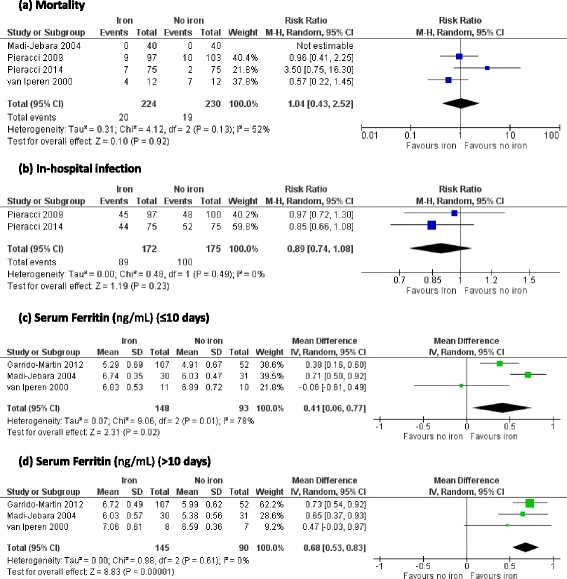
Effect of iron supplementation, by any route, on secondary outcomes **a** mortality **b** in-hospital infection and serum ferritin at **c** short-term follow-up and **d** medium-term follow-up. *CI* confidence interval, *M-H* Mantel-Haenszel test, *IV* inverse variance

Four trials reported in-hospital mortality as an outcome [21–24]. In meta-analysis there was no difference in the risk of mortality between patients who received iron supplementation and those who did not (RR 1.04, 95 % CI 0.43 to 2.52; *p* = 0.92, *I*^2^ = 52 %) (Fig. 5a). Causes of mortality were not reported in any trials.

### In-hospital infection

Two studies provided data on infection and antibiotic usage [22, 23]. There was no evidence of a difference in the risk of infection in patients who received iron supplementation and those who did not (RR 0.89, 95 % CI 0.74 to 1.08, *p* = 0.23, *I*^2^ = 0 %) (Fig. 5b). Neither study found a significant difference in mean number of antibiotic days between the intervention and control groups (14 vs. 16 days, *p* = 0.45; 14 vs. 16 days, *p* = 0.64).

### Length of stay

Three trials reported ICU LOS [22–24] and two trials additionally reported hospital LOS [22, 23]. One trial reported a significantly longer ICU LOS in the control group compared to the intervention group (58 +/- 31 vs. 29 +/- 18 days, *p* < 0.05) [24], whereas two trials found no difference in ICU LOS (12 vs. 14 days, *p* = 0.69; 10 vs. 11 days, *p* = 0.53) [22, 23]. Similarly, these trials found no differences in hospital LOS (14 vs. 16 days, *p* = 0.24; 14 vs. 16 days, *p* = 0.50) [22, 23]. Meta-analysis was not performed as only one trial provided mean and SD values.

### Biomarkers of iron status

All trials reported serum ferritin measurements. Three trials reported mean and SD data [19, 21, 24] which were log-transformed due to the likely skewing of this outcome. Iron supplementation was associated with higher log serum ferritin levels at both short-term (MD 0.41 ng/mL, 95 % CI 0.06 to 0.77, *p* = 0.02, *I*^2^ = 78 %) (Fig. 5c) and mid-term follow up (MD 0.68 ng/mL, 95 % CI 0.53 to 0.83, *p* < 0.00001, *I*^2^ = 0 %) (Fig. 5d). In two trials that did not report mean and SD values, in one trial of oral iron supplementation there was no difference in serum ferritin between iron and placebo groups throughout the study period [22] and in one trial of intravenous iron supplementation there were significantly higher serum ferritin levels in the iron group compared to the placebo group at day 7 (median 808 ng/mL vs. 457 ng/mL, *p* < 0.01) and day 14 (median 1,046 ng/mL vs. 551.5 ng/mL, *p* < 0.01) [23].

Three trials reported serum iron and eZPP measurements [22–24], all of which found no significant difference in concentrations between iron and placebo groups at any time point. Meta-analysis was precluded by a lack of reporting of mean and SD values. Two trials reported Tsat levels – in one trial there was a statistically significant increase in Tsat levels at day 7 in the intravenous iron group when compared to placebo (15 % vs. 11 %, respectively, *p* = 0.02) but this was below the normal range and did not persist through to the end of the study period [21], whereas in another trial there were no differences in Tsat levels throughout the study period [24].

### Other secondary outcomes

No trials reported data on morbidity scales, HrQoL scores or serious adverse events as per the definition above.

### Adverse events

Only one trial [22] comparing oral iron to placebo reported adverse effects - namely gastrointestinal upset defined as “nausea, vomiting, abdominal cramping, or constipation, necessitating discontinuation of the study drug for any period of time”. The authors found no difference between the iron and placebo groups (12.4 % vs. 8.7 % respectively, *p* = 0.62).

## Discussion

This systematic review identified five randomised trials enrolling a total of 665 randomised participants to evaluate the effect of iron as a treatment for anaemia in critical care. There was no evidence that iron supplementation to treat anaemia in adult critical care reduced allogeneic RBC transfusion requirements or increased mean haemoglobin concentrations, and there was no evidence of an impact on mortality and length of stay. Serum ferritin concentrations appeared to be significantly elevated in patients who receive iron supplementation at both short-term and mid-term follow up. No studies reported on HrQoL. However, the available evidence from randomised trials of iron supplementation in critical care patients is incomplete, and the confidence intervals for all outcomes were wide, and these limits could encompass clinically important differences. Only one trial was graded at low-risk across all domains.

Across all outcomes the analyses in our review showed moderate heterogeneity between studies due to differences in patient population, the intervention and the intervention dose. The patient populations varied across all trials - cardiac, general surgical, trauma, neurosurgical and medical - limiting the external generalizability of the data. The majority of the patients included were in surgical ICUs. There was widespread variation in the dosing regimens of iron and methods of administration. The effect of oral iron may be reduced due to poor absorption in patients with impaired gastro-intestinal function – particularly in surgical/trauma patients. Intravenous iron may be more effective than oral iron in states of inflammation, as it can bypass the effects of hepcidin, an inhibitor of enteric absorption [11], but the four trials evaluating intravenous iron tested different formulations and schedules. Finally, the follow-up time points in all included studies were relatively short - the longest duration was up to 42 days, so any potential long-term clinical benefits of iron therapy may have been missed.

To our knowledge, this is the first systematic review of iron therapy in adult critical care. Strengths include a comprehensive literature search repeated at two time points, duplicate data extraction, and consideration of a broad range of biochemical and clinical outcomes. A multicentre, phase IIb, randomised, placebo-controlled trial in critically ill patients, comparing intravenous iron in addition to standard care with standard care alone is currently recruiting (ACTRN 12612001249842) [25], and will be incorporated into updated versions of this review.

Our systematic review should be interpreted alongside other recent reviews. However, these reviews provide little data specific to iron supplementation in critically ill patients. For example, a recent meta-analysis on the safety and efficacy of intravenous iron in reducing requirement for allogeneic blood transfusion across all patient groups suggests iron is effective in reducing the requirement for allogeneic RBC transfusion and increasing haemoglobin concentration, but this meta-analysis did not report subgroup analyses in critical care patients. The apparent benefit of iron reported in this review was also counterbalanced by an increased risk of infection [11]. There was no difference, however, in serious adverse events or mortality in those receiving intravenous iron compared with oral or no iron. Similarly, a systematic review [26] investigating the safety and efficacy of iron therapy (oral and/or intravenous) for the treatment of anaemia in adults (including critical care patients but without subgroup analysis) found that both oral and intravenous iron reduced the need for blood transfusion. Intravenous iron was more effective at improving haemoglobin levels and quality of life, but neither route reduced mortality.

Any potential advantages of iron therapy must be balanced against the risk of adverse events. The risk of infection with use of iron is a significant concern in critically ill patients. Free iron has been shown to promote bacterial growth in vitro [27] and a meta-analysis [11] reported that intravenous iron was associated with a significant risk of infection. In our review only one trial reported on adverse events, and two trials reported on infection outcomes, with no significant differences. The two trials included in this study that reported infection outcomes used the same standardized definitions for nosocomial infection. However, infection is often not an agreed predefined endpoint in many studies [11], which can limit the strength of the conclusions.

The characterisation of anaemia in critical care patients is currently challenging as the tests routinely used to screen for iron deficiency, such as ferritin and transferrin, are confounded by the presence of inflammation. The recent discovery of the hormone hepcidin, a key regulator of iron metabolism and inhibitor of enteric iron absorption, has opened up avenues for improved diagnosis and management of anaemia in patients with concomitant inflammation such as those with critical illness [28]. It has been postulated that hepcidin may provide a better marker of iron deficiency than the routine biochemical tests in current use, thereby allowing more precise identification of patients with anaemia who likely to respond to iron therapy, whether intravenous or oral. The concept of using serum hepcidin to identify iron deficiency in the context of inflammation has been successfully applied to other patient populations where it has also predicted responsiveness to iron therapy [29, 30]. Whether this holds true for critically ill patients requires further investigation. Furthermore, an understanding of the biological role of hepcidin in iron mobilisation might suggest that intravenous iron may be more advantageous as part of a targeted strategy to manage the iron-deficiency component of anaemia in critically ill patients, in whom serum hepcidin would be elevated and oral iron absorption therefore suppressed.

## Conclusion

This review has identified five small trials in a diverse group of critically ill patients. On the basis of low-moderate quality evidence, we have shown that there is no evidence that iron supplementation, either intravenous or enteral, to treat anaemia in critically ill patients reduces transfusion requirements or the mean number of RBCs transfused, or increases haemoglobin concentration. However the strength of this conclusion is limited by heterogeneity between studies and risks of bias. It is clear that further well-designed prospective studies in larger numbers of patients, with clinically relevant outcomes and taking into account many of the limitations identified by this review, are required to understand the role of iron supplementation in critical care. At present, the findings do not support any specific strategy for routine administration of iron, by any route, to treat in patients with anaemia in adult critical care.

## Additional file

Additional file 1: Search strategy. Search narrative first performed in March 2015 and repeated on 9 March 2016. (DOCX 37 kb)

## Abbreviations

CI: confidence interval
EPO: erythropoietin
eZPP: erythrocyte zinc protoporphyrin
HDU: high-dependency unit
HrQoL: health-related quality of life
ICU: intensive care unit
Iv: intravenous
LOS: length of stay
MD: mean difference
MODS: multiple organ dysfunction score
PRISMA: preferred reporting items for systematic reviews and meta-analyses
RBC: red blood cell
RCT: randomized controlled trial
SD: standard deviation
SOFA: sequential organ failure assessment
Tsat: transferrin saturation
