# Supplementary material for: Iron supplementation to treat anaemia in adult critical care patients: a systematic review and meta-analysis
Source: Crit Care. 2016 Sep 29;20:306. doi: 10.1186/s13054-016-1486-z (PMC5041556; doi:10.1186/s13054-016-1486-z)
Supplement: Additional file 1: — Search strategy. Search narrative first performed in March 2015 and repeated on 9 March 2016. (DOCX 37 kb) [file 13054_2016_1486_MOESM1_ESM.docx]

**ORAL OR PARENTERAL IRON FOR ANAEMIA IN CRITICAL CARE**

**SEARCH NARRATIVE, MARCH 2015 – MARCH 2016**

The following databases were searched on 9.3.16 for RCTs and SRs:

CENTRAL (*The Cochrane Library* Issue 2, 2016)
MEDLINE (1946 onwards)
EMBASE (last 6 months)
CINAHL (1982 onwards)
PUBMED (epublications only)
TRANSFUSION EVIDENCE LIBRARY (1950 onwards)
WEB OF SCIENCE (ISI Conference Proceedings)

Ongoing Trial Databases:
ClinicalTrials.gov
WHO International Clinical Trials Registry Platform (ICTRP)

**SEARCH STRATEGIES**

**CENTRAL**
#1 MeSH descriptor: [Intensive Care Units] explode all trees
#2 MeSH descriptor: [Critical Care] explode all trees
#3 MeSH descriptor: [Critical Illness] this term only
#4 MeSH descriptor: [Critical Care Nursing] this term only
#5 MeSH descriptor: [Trauma Centers] this term only
#6 ((critical* or serious* or severe*) near/2 (ill* or injur* or wound*))
#7 ((intensive* or critical* or neurointensive* or neurocritical*) near/3 (care or therap* or treatment*))
#8 (critical* or intensive* or trauma*):so
#9 (ICU or PICU or MICU or CICU or CVICU or CCU or NICU or SICU or POCCU or ITU or HDU)
#10 "high dependency" or "coronary care unit*"
#11 #1 or #2 or #3 or #4 or #5 or #6 or #7 or #8 or #9 or #10
#12 MeSH descriptor: [Iron] this term only
#13 MeSH descriptor: [Iron Compounds] this term only
#14 MeSH descriptor: [Ferric Compounds] explode all trees
#15 MeSH descriptor: [Ferrous Compounds] explode all trees
#16 MeSH descriptor: [Iron Carbonyl Compounds] this term only
#17 (iron or ferritin or ferrous):ti
#18 (ferrous next (sulfate* or sulphate* or saccharate* or succinate* or fumarate* or fumerate* or gluconate*)):ab
#19 "ferric gluconate" or Fersaday* or Galfer* or Ironorm* or Feospsan* or Ferrograd* or "sodium feredetate" or Sytron* or Niferex*
#20 (iron near/3 (oral* or supplement* or salt* or complex or inject* or infus* or intravenous* or IV or parenteral* or replac* or product* or tablet* or pill* or capsule* or sulphate* or sulfate* or saccharate* or gluconate* or therap*)):ab
#21 "iron sucrose" or venofer* or "iron dextran" or cosmofer* or "iron isomaltoside" or monofer* or ferumoxytol or rienso* or "ferric carboxymaltose" or ferinject*
#22 (dextriferron or fedex or "ferrum lek" or injectafer or "sodium ferrigluconate" or "ferric gluconate" or ferlecit or ferlixit or (ferric near/2 mattol))
#23 dextrofer or hematran or imposil or norferan or imferon or imperon or dexferrum or feosol or imfergen or ferridextran
#24 #12 or #13 or #14 or #15 or #16 or #17 or #18 or #19 or #20 or #21 or #22 or #23
#25 #11 and #24

**MEDLINE (OvidSP)**
1. exp Intensive Care Units/

2. exp Critical Care/

3. Critical Illness/

4. Critical Care Nursing/

5. Trauma Centers/

6. ((critical* or serious* or severe*) adj2 (ill* or injur* or wound*)).tw.

7. ((intensive* or critical* or neurointensive* or neurocritical*) adj3 (care or therap* or treatment*)).tw.

8. (critical* or intensive* or trauma*).jn.

9. (ICU or PICU or MICU or CICU or CVICU or CCU or NICU or SICU or POCCU or ITU or HDU).tw.

10. (high dependency or coronary care unit*).tw.

11. or/1-10

12. Iron/

13. Iron Compounds/

14. exp Ferric Compounds/

15. exp Ferrous Compounds/

16. Iron Carbonyl Compounds/

17. (iron or ferritin or ferrous).ti.

18. (ferrous adj (sulfate* or sulphate* or saccharate* or succinate* or fumarate* or fumerate* or gluconate*)).ab.

19. (ferric gluconate or Fersaday* or Galfer* or Ironorm* or Feospsan* or Ferrograd* or sodium feredetate or Sytron* or Niferex*).tw.

20. (iron adj3 (oral* or supplement* or salt* or complex or inject* or infus* or intravenous* or IV or parenteral* or replac* or product* or tablet* or pill* or capsule* or sulphate* or sulfate* or saccharate* or gluconate* or therap*)).ab.

21. (iron sucrose or venofer* or iron dextran or cosmofer* or iron isomaltoside or monofer* or ferumoxytol or rienso* or ferric carboxymaltose or ferinject*).tw.

22. (dextriferron or fedex or ferrum lek or injectafer or sodium ferrigluconate or ferric gluconate or ferlecit or ferlixit or (ferric adj2 mattol)).tw.

23. (dextrofer or hematran or imposil or norferan or imferon or imperon or dexferrum or feosol or imfergen or ferridextran).tw.

24. or/12-23

25. 11 and 24

**EMBASE (OvidSP)**

1. exp Intensive Care/

2. Intensive Care Unit/

3. Coronary Care Unit/

4. Burn Unit/

5. Stroke Unit/

6. Critical Illness/

7. ((critical* or serious* or severe*) adj2 (ill* or injur* or wound*)).tw.

8. ((intensive* or critical* or neurointensive* or neurocritical*) adj3 (care or therap* or treatment*)).tw.

9. (critical* or intensive* or trauma*).jn.

10. (ICU or PICU or MICU or CICU or CVICU or CCU or NICU or SICU or POCCU or ITU or HDU).tw.

11. (high dependency or coronary care unit*).tw.

12. or/1-11

13. antianemic agent/ or chondroitin sulfate iron/ or dextriferron/ or ferric carboxymaltose/ or ferric gluconate/ or ferric maltol/ or ferric pyrophosphate/ or ferrocholinate/ or ferrous fumarate/ or ferrous gluconate/ or ferrous succinate/ or ferrous sulfate/ or ferrous sulfate plus folic acid/ or ferrous sulfate plus multivitamin/ or ferumoxytol/ or iron dextran/ or iron glycinate/ or iron polymaltose/ or iron polysaccharide/ or iron protein succinylate/ or iron saccharate/ or iron salt/ or iron sorbitex/ or resoferon/

14. *Iron/

15. Iron Therapy/

16. Ferric Ion/

17. Ferrous Iron/

18. (iron or ferritin or ferrous).ti.

19. (ferrous adj (sulfate* or sulphate* or saccharate* or succinate* or fumarate* or fumerate* or gluconate*)).ab.

20. (ferric gluconate or Fersaday* or Galfer* or Ironorm* or Feospsan* or Ferrograd* or sodium feredetate or Sytron* or Niferex*).tw.

21. (iron adj3 (oral* or supplement* or salt* or complex or inject* or infus* or intravenous* or IV or parenteral* or replac* or product* or tablet* or pill* or capsule* or sulphate* or sulfate* or saccharate* or gluconate* or therap*)).ab.

22. (iron sucrose or venofer* or iron dextran or cosmofer* or iron isomaltoside or monofer* or ferumoxytol or rienso* or ferric carboxymaltose or ferinject*).tw.

23. (dextriferron or fedex or ferrum lek or injectafer or sodium ferrigluconate or ferric gluconate or ferlecit or ferlixit or (ferric adj2 mattol)).tw.

24. (dextrofer or hematran or imposil or norferan or imferon or imperon or dexferrum or feosol or imfergen or ferridextran).tw.

25. or/13-24

25. 12 and 25

**CINAHL (EBSCOHost)**

S1 (MH "Critical Care+")

S2 (MH "Intensive Care Units+")

S3 (MH "Critical Illness")

S4 (MH "Catastrophic Illness")

S5 (MH "Critically Ill Patients")

S6 (MH "Critical Care Nursing+")

S7 (MH "Trauma Centers")

S8 TX ((critical* or serious* or severe*) N2 (ill* or injur* or wound*))

S9 TX ((intensive* or critical* or neurointensive* or neurocritical*) N3 (care or therap* or treatment*))

S10 JN (critical* or intensive* or trauma*)

S11 TX (ICU or PICU or MICU or CICU or CVICU or CCU or NICU or SICU or POCCU or ITU or HDU)

S12 TX ("high dependency" or "coronary care unit*")

S13 S1 OR S2 OR S3 OR S4 OR S5 OR S6 OR S7 OR S8 OR S9 OR S10 OR S11 OR S12

S14 (MH "Iron")

S15 (MH "Iron Compounds+")

S16 TI  (iron or ferritin or ferrous)

S17 AB (ferrous W1 (sulfate* or sulphate* or saccharate* or succinate* or fumarate* or fumerate* or gluconate*))

S18 TX ("ferric gluconate" or Fersaday* or Galfer* or Ironorm* or Feospsan* or Ferrograd* or sodium feredetate or Sytron* or Niferex)

S19 TX (iron N3 (oral* or supplement* or salt* or complex or inject* or infus* or intravenous* or IV or parenteral* or replac* or product* or tablet* or pill* or capsule* or sulphate* or sulfate* or saccharate* or gluconate* or therap*))

S20 TX ("iron sucrose" or venofer* or "iron dextran" or cosmofer* or "iron isomaltoside" or monofer* or ferumoxytol or rienso* or "ferric carboxymaltose" or ferinject*)

S21 TX (dextriferron or fedex or "ferrum lek" or injectafer or "sodium ferrigluconate" or "ferric gluconate" or ferlecit or ferlixit or (ferric W2 mattol))

S22 TX (dextrofer or hematran or imposil or norferan or imferon or imperon or dexferrum or feosol or imfergen or ferridextran)

S23 S14 OR S15 OR S16 OR S17 OR S18 OR S19 OR S20 OR S21 OR S22

S24 S13 AND S23

**PubMed (epublications)**

#1 ((critical* OR serious* OR severe*) AND (ill OR illness* OR injur* OR wound*))

#2 ((intensive* OR critical* OR neurointensive* OR neurocritical*) AND (care OR therapy OR therapies OR treatment*))

#3 (ICU OR PICU OR MICU OR CICU OR CVICU OR CCU OR NICU OR SICU OR POCCU OR ITU OR HDU)

#4 "high dependency" OR "coronary care unit"

#5 critical*[TI] OR seriously[TI] OR severely[TI] OR intensive*[TI] OR neurointensive*[TI] OR neurocritical*[TI]

#6 #1 OR #2 OR #3 OR #4 OR #5

#7 iron[TI] OR ferritin[TI] OR ferrous[TI]

#8 ("ferrous sulfate" OR "ferrous sulphate" OR "ferrous saccharate" OR "ferrous fumarate" OR "ferrous fumerate" OR "ferrous gluconate" OR "ferrous succinate")

#9 ("ferric gluconate" OR Fersaday* OR Galfer* OR Ironorm* OR Feospsan* OR Ferrograd* OR "sodium feredetate" OR Sytron* OR Niferex*)

#10 ("oral iron" OR "iron supplement" OR "iron supplements" OR "iron supplementation" OR "iron salt" OR "iron salts" OR "iron complex" OR "injectable iron" OR "iron infusion" OR "iron infusions" OR "infused iron" OR "intravenous iron" OR "IV iron" OR "parenteral iron" OR "iron replacement" OR "iron product" OR "iron products" OR "iron tablet" OR "iron tablets" OR "iron pill" OR "iron pills" OR "iron capsule" OR "iron capsules" OR "iron sulphate" OR "iron sulfate" OR "iron saccharate" OR "iron gluconate" OR "iron therapy")

#11 ("iron sucrose" OR venofer* OR "iron dextran" OR cosmofer* OR "iron isomaltoside" OR monofer* OR ferumoxytol OR rienso* OR ferric carboxymaltose OR ferinject*)

#12 (dextriferron OR fedex OR ferrum lek OR injectafer OR "sodium ferrigluconate" OR "ferric gluconate" OR ferlecit OR ferlixit OR "ferric mattol")
#13 (dextrofer or hematran or imposil or norferan or imferon or imperon or dexferrum or feosol or imfergen or ferridextran)

#14 #7 OR #8 OR #9 OR #10 OR #11 OR #12 OR #13

#15 #6 AND #14

#16 (random* OR blind* OR "control group" OR placebo* OR "controlled trial" OR "controlled study" OR groups OR trials OR studies OR "systematic review" OR meta-analysis OR metaanalysis OR "literature search*" OR medline OR cochrane OR embase) AND (publisher[sb] OR inprocess[sb] OR pubmednotmedline[sb])
#17 #15 AND #16

**TRANSFUSION EVIDENCE LIBRARY**
Clinical Specialty: Critical Care
Subject Area: Management of Anaemia
AND
iron OR ferrous OR ferric

**WEB OF SCIENCE – Conference Proceedings Citation Index (CPCI-S)**#1 TS=(critical care OR intensive care OR critically ill OR critical illness OR trauma)
#2 TS=(iron OR ferritin OR ferrous OR ferric)
#3 TS=(randomized OR randomised OR randomly OR controlled trial OR controlled study OR control group OR control groups OR double blind)
#4 #1 AND #2 AND #3

**WHO ICTRP**
Title: critical OR critically OR intensive OR intensively OR serious OR seriously OR ICU OR PICU OR MICU OR CICU OR CVICU OR CCU OR NICU OR SICU OR POCCU OR ITU OR HDU OR high dependency OR unit
Condition: iron deficiency OR iron deficient OR low iron OR anemia OR anaemia OR low hemoglobin OR low haemoglobin OR low red cell count
Intervention: iron OR ferritin OR ferrous OR ferric
Recruitment: ALL

**ClinicalTrials.gov**Search Terms: critical OR critically OR intensive OR intensively OR serious OR seriously OR ICU OR PICU OR MICU OR CICU OR CVICU OR CCU OR NICU OR SICU OR POCCU OR ITU OR HDU OR high dependency OR unit
Condition: iron deficiency OR iron deficient OR low iron OR anemia OR anaemia OR low hemoglobin OR low haemoglobin OR low red cell count
Intervention: iron OR ferritin OR ferrous OR ferric
Type of Studies: Interventional
